# A randomized trial and novel SPR technique identifies altered lipoprotein-LDL receptor binding as a mechanism underlying elevated LDL-cholesterol in *APOE4s*

**DOI:** 10.1038/srep44119

**Published:** 2017-03-09

**Authors:** M. V. Calabuig-Navarro, K. G. Jackson, C. F. Kemp, D. S. Leake, C. M. Walden, J. A. Lovegrove, A. M. Minihane

**Affiliations:** 1 Hugh Sinclair Unit of Human Nutrition, Department of Food & Nutritional Sciences, University of Reading, Reading RG6 6LA, United Kingdom; 2 Institute for Cardiovascular and Metabolic Research (ICMR), University of Reading, Reading RG6 6LA, United Kingdom; 3 Biocentre, University of Reading, Reading RG6 6AP United Kingdom; 4 Unilever Discover, Colworth Science Park, Sharnbrook, Bedford, MK44 1LQ, United Kingdom

## Abstract

At a population level *APOE4* carriers (~25% Caucasians) are at higher risk of cardiovascular diseases. The penetrance of genotype is however variable and influenced by dietary fat composition, with the *APOE4* allele associated with greater LDL-cholesterol elevation in response to saturated fatty acids (SFA). The etiology of this greater responsiveness is unknown. Here a novel surface plasmon resonance technique (SPR) is developed and used, along with hepatocyte (with the liver being the main organ modulating lipoprotein metabolism and plasma lipid levels) uptake studies to establish the impact of dietary fatty acid composition on, lipoprotein-LDL receptor (LDLR) binding, and hepatocyte uptake, according to *APOE* genotype status. In men prospectively recruited according to *APOE* genotype (*APOE3/3* common genotype, or *APOE3/E4*), triglyceride-rich lipoproteins (TRLs) were isolated at fasting and 4–6 h following test meals rich in SFA, unsaturated fat and SFA with fish oil. In *APOE4s* a greater LDLR binding affinity of postprandial TRL after SFA, and lower LDL binding and hepatocyte internalization, provide mechanisms for the greater LDL-cholesterol raising effect. The SPR technique developed may be used for the future study of the impact of genotype, and physiological and behavioral variables on lipoprotein metabolism. Trial registration number NCT01522482.

Although recent meta-analyses suggest no overall association between saturated fat (SFA) intake and cardiovascular disease (CVD) incidence or mortality^1^, the LDL-cholesterol lowering effects of the replacement of dietary SFA with unsaturated fatty acids is well established^2^,^3^,^4^. Individuals with an *APOE4* genotype (~25% Caucasians) are particularly sensitive^5^,^6^ with this responsiveness providing *APOE4* carriers with a tractable means of reducing the penetrance of this ‘at-risk’ genotype. A 20–30% reduction in population SFA intake in Western Countries since the 1970s^7^ may go a long way towards explaining the apparent attenuation in reported relative risk of coronary heart disease associated with the *APOE4* allele in recent decades^8^,^9^,^10^,^11^. However although repeatedly observed, an understanding of the mechanistic basis for the greater sensitivity to dietary fat composition in *APOE4* carriers is lacking. Such biological plausibility would provide justification for the targeting of *APOE4* individuals as a large population subgroup who would particularly benefit from reduced SFA intakes.

ApoE, found in the circulation on the surface of high density lipoproteins (HDL) and triglyceride-rich lipoproteins (TRL, chylomicrons (CM), VLDL and their remnants) is involved in lipoprotein production, and hepatic clearance from the circulation by acting as a high affinity ligand for the LDL receptor (LDLR) family^5^,^12^. Unlike the cholesterol-rich VLDL2 and LDL which mainly use apoB-100, apoE acts as the primary ligand for the uptake of triglyceride-rich VLDL1^13^. A competition study performed *in vitro* showed that the uptake of LDL by HepG2 (liver) cells is reduced in the presence of apoE-containing TRL predominately in the VLDL1 density range^14^. A greater enrichment of the TRL particles with apoE after a SFA-rich meal reduced the HepG2 LDL uptake relative to TRL isolated after meals containing unsaturated fatty acids^14^. A more recent human *ex vivo* investigation has shown that smaller TRL (predominately VLDL2) isolated from *APOE4* carriers reduced the uptake of LDL compared with the wild-type *APOE3/E3* group^15^. Therefore a greater competition between TRL isolated from *E4* carriers with LDL for uptake by the LDLR may provide an explanation for the higher fasting LDL-cholesterol and in particular against a high SFA background diet. However, such ‘competition’ studies based on the uptake of ^125^I-labelled LDL do not provide a holistic overview of lipoprotein metabolism focusing only on the fate of the LDL particles, with no information about the binding properties of TRL with the LDLR and subsequent hepatocyte internalization.

Surface plasmon resonance (SPR), is a sensor based technology that allows the interaction of biological molecules to be determined in real-time^16^,^17^. The technique has previously been used to investigate the binding of purified apoE and apoB to lipoprotein particles^18^ or to specific receptors^12^,^19^,^20^,^21^. However, little is known about the kinetics of the more physiologically relevant scenario of the binding of intact lipoprotein particles (containing apoB and apoE) to the LDLR. Thus the initial aim of this *ex vivo* study series was to develop a SPR technique for use in the study of lipoprotein metabolism and specifically the binding of lipoproteins to the LDLR. This was then applied, together with uptake studies in a HepG2 cell model to investigate the effects of *APOE* genotype and meal fat composition on the *ex vivo* binding of TRL and LDL to the LDLR and hepatic lipoprotein uptake. In addition to SFA and PUFA we investigated the impact of the fish derived n-3 fatty acid docosahexaenoic acid (DHA) on lipoprotein binding. The LDL-cholesterol raising effect of high dose DHA has been repeatedly described^22^ with inconsistent evidence indicating this may be partly *APOE* genotype dependent^15^,^23^,^24^.

## Materials and Methods

### Subjects and study design

The subject group, test meal composition and postprandial study day are described in detail in a previous publication^25^. Briefly n = 31 healthy men (mean ± SD, age 48 ± 16 y, BMI 25.7 ± 2.7 kg/m^2^) were prospectively recruited according to *APOE* genotype in Reading, UK, from the University of Reading and surrounding area between January 2009 and April 2009, with the intervention completed by Dec 2009. Participants underwent a single blind randomized cross-over trial, consuming on 3 separate occasions a mixed meal containing 53 g of fat, of which 50 g was provided in the form of a test oil rich in either SFA (29 g palm oil and 21 g cocoa butter), unsaturated fatty acids (UNSAT, 7.2 g rapeseed oil, 16.2 g soybean oil and 26.6 g olive oil) or SFA with fish oils (SFA-FO, 22 g palm oil, 18 g cocoa butter and 10 g fish oil, DHA:EPA 7.3:1), in random order. The order of intervention was decided by the trial statistician based on simple randomization and a web-based random letter sequence generator. *APOE3/E3* (n = 10) and *APOE3/E4* (n = 11) completed all three intervention arms.

Blood samples were collected at fasting and 180, 240, 300, 360 and 480 min after the test meals. The study and all experimental protocols were approved by the University of Reading Research Ethics Committee. All methods were conducted according to the guidelines in the Declaration of Helsinki. All participants gave their written informed consent before commencing the study. This trial was registered at clinicaltrials.gov as NCT01522482 (26^th^ January 2012).

### Preparation of lipoprotein fractions for SPR analysis and HepG2 cell studies

Blood for LDL isolation was obtained from an independent group of healthy *APOE3/E3* and *APOE3/E4* male subjects after an overnight fast. LDL (density 1.019–1.063 g/ml) was isolated as described previously^26^. A portion of the LDL fraction from each genotype group was radiolabeled using iodogen^27^.

TRL fractions, Svedberg flotation rate (S_f_) 60–400 (contains predominately VLDL1 particles, and so this fraction will be referred to in the remainder of the paper as VLDL1-rich) and S_f_ 20–60 (VLDL2-rich) were isolated from both fasting (0 min) and postprandial plasma^28^. The time point chosen for the postprandial sample for *in vitro* studies represented the individual’s peak VLDL1-rich fraction apoB and apoE concentration (usually between 240–360 min) observed after each meal in our human study^25^. In order to ensure a sufficient yield of apoB containing lipoproteins in the VLDL1-rich and VLDL2-rich TRL fractions, the samples were pooled from two subjects before desalting through PD-10 desalting columns (Amersham Biosciences, Chalfont St. Giles, UK) and further concentrated using Vivaspin concentrators (Sartorius Ltd, Epsom, UK). Since different salt solutions were used for the isolation of the TRL fractions and LDL, the VLDL1-rich and VLDL2-rich fractions were further dialyzed against 0.02 M phosphate buffer containing 100 μM EDTA for 24 h at 4 °C to minimize the effects of the salt solution on the binding signal during the SPR analysis. Apos B and E were measured using an ILAB600 clinical chemistry analyzer (Instrumentation Laboratory, Warrington, UK) by turbidimetric immunoassay (Kamiya Biomedical, Seattle, USA).

### Surface plasmon resonance (SPR)

**Figure 1:**
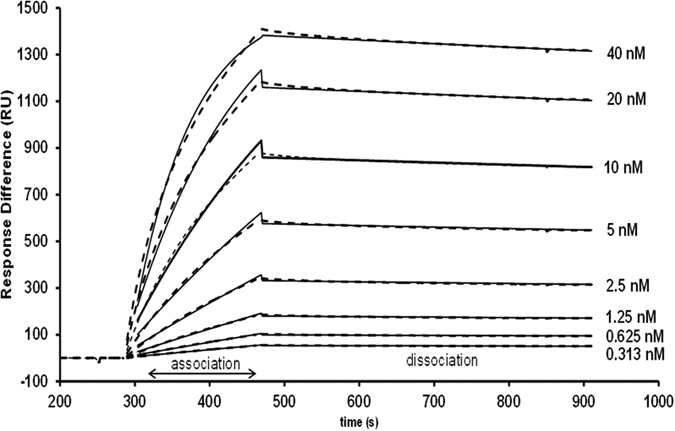
Sensorgram of the interaction between VLDL-1 rich particles (ranging from 0 to 40 nM apoB) and the LDL-receptor immobilized on the surface of a CM5 sensor chip. Experimental data (dotted lines) was fitted with the two-state binding model (solid line).

The binding affinity of the lipoproteins to the LDLR receptor, was investigated with a BIAcore 3000 SPR instrument (GE Healthcare, Little Chalfont, UK) using a CM5 sensor chip (GE Healthcare). This technique measures changes in the refractive index near the sensor surface^29^, that is proportional to the mass of the analyte when it binds to the ligand (generally a receptor) immobilized on the chip surface. This change in refractive index is monitored in real time and is displayed as a sensorgram (see Fig. 1)^30^,^31^,^32^.

Purified LDLR ectodomain (R & D Systems, Abingdon, UK) diluted with acetate buffer (pH 5.0; GE Healthcare) to a final concentration of 10 μg/ml, was immobilized onto the surface of a CM5 sensor chip with a target of 1000 resonance units (RU) by amine coupling in accordance with the manufacturer’s instructions (GE Healthcare). A flow cell on the chip without the addition of LDLR was used as a control. The final step involved deactivation of the excess reactive groups on the surface of the CM5 sensor chip using 1 M ethanolamine-HCl (pH 8.5) to prevent non-specific binding of the lipoproteins to the chip surface.

The concentrated lipoprotein fractions (VLDL1-rich, VLDL2-rich and LDL) were diluted to a final concentration of 5 nM apoB in filtered running buffer (HBS-N 1X (pH 7.4, 0.01 M HEPES, 0.15 M sodium chloride, GE Healthcare)) containing 0.5% BSA (w/v) and 1 mM calcium chloride. The diluted samples were filtered through 0.22 μm filters (Millipore, USA) before injection to remove aggregated proteins that could interfere with LDLR binding. To ensure that binding of the lipoprotein fractions to the LDLR was not influenced by mass transfer effects (i.e. size of the lipoprotein particles) all binding experiments were performed at 25 °C using a flow rate of 30 μl/min for 3 min. Following dissociation of the lipoprotein particles from the LDLR, the sensor chip surface was regenerated with 7.5 mM sodium hydroxide in 100 mM EGTA. Individual lipoprotein samples (LDL, VLDL1-rich or VLDL2-rich TRL) were injected in duplicate and two blanks (running buffer) were injected every 10 samples to decrease the loss of activity of the chip surface. A control VLDL1-rich sample was injected at the beginning and at the end of each experiment to assess for both inter- and intra-assay variation and to ensure the functionalized surface (LDLR) retained sufficient binding activity throughout the analysis period.

The sensorgram data were analyzed with BIA evaluation 4.1 software (GE Healthcare). Sensorgram curves are displayed as RU versus *t*, where RU is the SPR signal response and *t* is time per second. For a CM5 chip, a response of 1 RU is equivalent to a change in surface concentration of LDLR of approximately 1 pg/mm^2^. The sensorgram data corresponding to the injection and post injection phases were used to perform the kinetic analysis to determine the best model fit for the binding of the lipoproteins to the LDLR. This was evaluated by calculating the goodness-of fit Chi-square distribution (Chi^2^) value, residual range and visual inspection of the fitted model.

### Cell culture and ^125^I-labelled LDL uptake studies

HepG2 cells (European Collection of Cell Culture, Centre for Applied Microbiology and Research, Salisbury, UK), were grown to 80% confluence in 24 well plates and maintained in charcoal-stripped lipoprotein-deficient serum (First Link UK, Birmingham, UK) for the 48 h prior to the addition of the lipoprotein fractions (LDL and TRL) to the medium, in order to upregulate LDLR on the cell surface.

Total LDL uptake which is the sum of the cell surface bound (heparin-releasable binding) and internalized ^125^I-LDL (cell associated radioactivity) including degradation products in the medium, was determined as described by Goldstein, Basu and Brown^33^ with a few modifications^14^. Briefly, ^125^I-labelled LDL at a final concentration of 10 μg protein/ml was added to the medium of HepG2 cells alone and simultaneously with 15 μg apoB/ml of VLDL1 or VLDL2-rich fractions for 5 hr at 37 °C. In all experiments, individual TRL fractions were incubated with ^125^I-labelled LDL from the same *APOE* genotype group. The results were expressed as a percentage relative to the incubation of the HepG2 cells with ^125^I-LDL alone.

### Statistical Analysis

Data were analyzed using SPSS software version 17.0 (SPSS Inc Chicago, USA). Results are presented as means ± SD or SEM. Data were checked for normality using the Shapiro-Wilk test and log-transformed where necessary. A Student’s independent t-test was used to determine differences in the baseline (fasting) binding properties of the lipoprotein samples to the LDLR and ^125^I-labelled LDL total uptake in the presence of fasting TRL fractions between genotype groups (*E3/E3* and *E3/E4*). For the binding properties of the postprandial TRL samples to the LDLR and HepG2 cell data, a mixed factor ANOVA with repeated measures was used, with test meal and genotype as the ‘within’ and ‘between’ factors respectively. A Bonferroni correction was used for the post hoc detection of significant pair wise differences. Values of P ≤ 0.05 were given to be statistically significant.

## Results

### Development of the SPR technique to study lipoprotein metabolism

**Figure 2:**
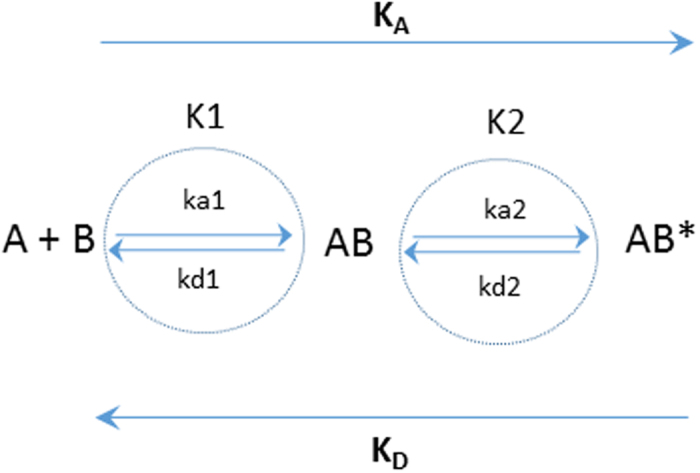
Binding of lipoproteins to the LDL-receptor. Lipoproteins (**A**) bind to the LDLR (**B**) to form complex AB, followed by a conformational change to form a more stable complex AB*. The association rate constants are ka1 and ka2 and the dissociation rate constants are kd1 and kd2. The equilibrium constant for each binding step are K1 = _  _ka1/kd1 and K2 = ka2/kd2. The overall equilibrium binding constants are therefore K_A_ = K1 (1 + K2) and K_D_ = 1/K_A_.

**Figure 3:**
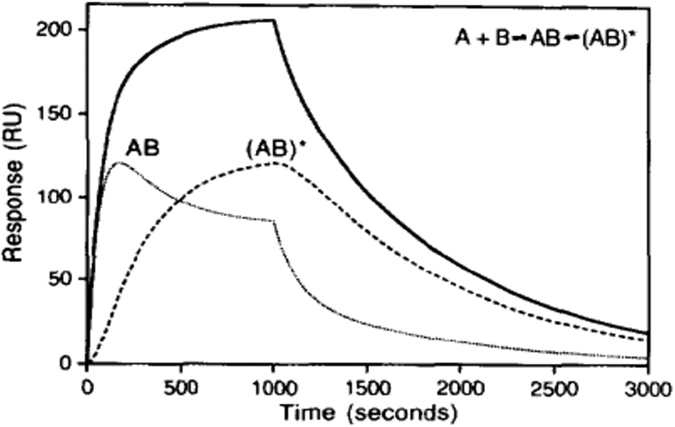
Overview of the sensorgrams for the two state conformational change reaction (A + B ↔ AB ↔ AB*). The output response (solid line) is the sum of the individual responses for the fast process to form AB and the conformational change to form the more stable complex AB*. Reprinted from Morton *et al*. (1985) with permission from the author^29^.

In order to study the binding affinities of different lipoprotein classes to the LDLR, LDL and TRL fractions were injected over a range of apoB concentrations (to normalize for particle number) to determine the concentration that resulted in the optimal binding signal to the LDLR. An example of a typical sensorgram showing the binding and dissociation of the VLDL-1 rich fraction particles over a range of apoB concentrations is given in Fig. 1. The final apoB concentration chosen for our subsequent SPR analysis was 5 nM; higher apoB concentrations deviated from the model (higher Chi^2^ values, data not shown) and lower concentrations were affected by the loss of activity of the chip surface after repeated regeneration conditions. For interpretation of the specificity of lipoprotein binding to the LDLR, the data were corrected for the bulk refractive index effect (associated with buffer washes). It was fitted using a two-state binding model since a lower Chi^2^ indicated that this was a better model fit compared with the 1:1 Langmuir binding model (data not shown). In this model (summarized in Fig. 2), lipoproteins (A) bind to the LDLR (B) to form complex AB, followed by a conformational change to form a more stable complex AB* (Fig. 3).

**Figure 4:**
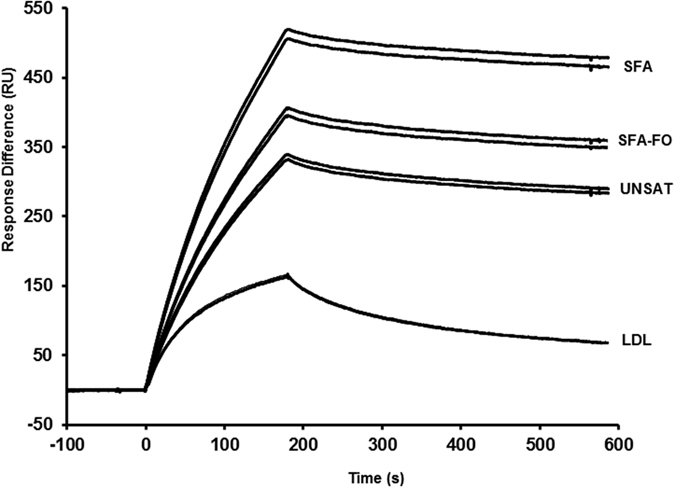
Effects of test meal composition on the binding of postprandial VLDL-1 rich TRL compared with fasting LDL particles to immobilized LDLR on a CM5 chip. The figure represents single pooled samples (from two study participants) post SFA, UNSAT and SFA-DHA meal consumption, run in duplicate. The lipoprotein fractions were normalized for lipoprotein particle number using apoB concentration. Abbreviations: SFA, saturated fatty acid meal; SFA-FO, SFA meal with fish oil; UNSAT, unsaturated fatty acid meal.

The binding of LDL to the LDLR was best described by the heterogeneous ligand model, as lower Chi^2^ values were observed compared with the two-state binding model (Chi^2^ = 0.412 vs 1.070, respectively). The differences in model-fits between LDL and TRL fractions can also be observed in the shape of the curves in the sensorgram (Fig. 4). However, the information that this model provides did not allow the direct comparison with the two state model used for the TRL samples. When the Chi^2^ values were compared between both models for LDL, no statistical differences were observed (*P* = 0.24 and *P* = 0.19 for LDL isolated from *APOE3/E3* and from *APOE3/E4* subjects, respectively). An additional comparison of F-values (F = 3.8e-6 for the heterogeneous ligand model, F = 9.9e-5 for the two state model), confirmed either were acceptable models to use, so the two state model was chosen for the analysis of all samples, as this would give the most directly comparable results, and allow for the identification of differences between the two interactions.

In order to establish variability, control VLDL1 sample were included in each run, which yielded intra- and inter-assay % CV of 5.3% (n = 8) and 9.7% (n = 5) for K_A_ values.

### APOE genotype affects the binding of fasting lipoproteins to the LDLR

Overall the global affinity (K_A_) of the different lipoprotein fractions for binding to the LDLR in the fasting state are in the following order VLDL1-rich TRL > VLDL2-rich TRL > LDL, which reflects differences in the formation of the stable AB* complex (ka2) which is faster with TRL and dissociates at a slower rate (kd2) relative to LDL.

*APOE* genotype impacted on the equilibrium constant with a lower K1 (*P* = 0.005) and K2 (*P* = 0.019) evident for LDL isolated from *APOE3/E4* compared with the *APOE3/E3* subjects (Table 1). This was reflected in the K_A_ (0.5 × 10^9^ M^−1^ vs. 0.7 × 10^9^ M^−1^, respectively) (*P* = 0.019) indicating that LDL isolated from *APOE3/E4* subjects had a lower binding affinity to the LDLR relative to the *APOE3/E3* genotype.

Relative to the *APOE3/E3* group, the dissociation rate constant (kd2) was lower (*P* = 0.013) and overall K2 was higher (*P* = 0.032) for the second step of the binding of fasting *APOE3/E4* VLDL1-rich particles to the LDLR. This indicates that once the AB^*^ complex is formed, it is more stable and dissociates at a slower rate after binding of fasting VLDL1-rich samples from *E3/E4* subjects to the LDLR. There were no significant genotype effects observed for the binding of the fasting VLDL2-rich fraction (Table 1), nor on the apoE content of either fasting TRL fraction estimated using the apoE/apoB ratio.

### ApoE content of the postprandial TRL particles

A significant meal effect was observed for the apoE/apoB ratio in the postprandial VLDL1- rich fraction (*P* = 0.023), with 2.9 ± 0.4, 2.9 ± 0.7 and 1.7 ± 0.5 apoE molecules per particle following the SFA, SFA-FO and UNSAT meal respectively. A tendency for an effect of meal fatty acid intake on the apoE/apoB ratio in the VLDL2-rich fraction was also evident with 2.1 ± 0.7, 1.4 ± 0.4 and 1.4 ± 0.3 apoE molecule per particle following the SFA, SFA-FO and UNSAT meals respectively (*P* = 0.100). No impact of genotype on the TRL apoE/apoB ratios were evident.

### *APOE* genotype and the binding of postprandial TRL fractions to the LDLR

There were no significant genotype or meal*genotype interactions observed for the binding of postprandial TRL fractions to the LDLR. Therefore, data for the two genotype groups were combined to determine the overall effect of the SFA, SFA-FO and UNSAT meals on the binding affinities of the postprandial TRL (Table 2). An almost 2-fold increase in K_A_ of the VLDL1-rich fraction to the LDLR was observed following the SFA relative to the SFA-FO or UNSAT meals (*P* ≤ 0.001; Fig. 3 and Table 2), which reflects a higher ka1 (*P* = 0.004), K1 (*P* = 0.007), and lower kd1 (*P* = 0.047).

Less marked effects of meal composition were observed for the VLDL2-rich fraction. Meal fat composition was shown to influence the rate of association of the AB* complex (ka2) (*P* = 0.011) (Table 2), indicating a higher rate of conversion of AB into the stable AB* complex with particles isolated following the SFA than UNSAT meal. Although the impact did not reach significance (*P* = 0.09), as observed in the VLDL1-rich fraction, the K_A_ for the VLDL2-rich fraction was in the order SFA > SFA-FO > UNSAT.

### Genotype and meal fat composition influence ^125^I-labelled LDL uptake on co-incubation with TRL

The concentration of the TRL samples used in the competition studies were normalized for the apoB content (15 μg apoB/ml) to control for particle number. In the fasting state a significant genotype effect was observed for the percentage of ^125^I-LDL bound to the surface of the HepG2 cells (heparin releasable binding), with a lower percentage binding in the presence of the VLDL1-rich (*P* = 0.001) and VLDL2-rich (*P* = 0.038) fractions isolated from *APOE3/E4* than *APOE3/E3* individuals (Table 3). Genotype effects were not observed for the percentage cell associated radioactivity (internalized), degradation products (released into the medium) or total uptake of ^125^I-LDL following co-incubation with the fasting TRL fractions.

In the postprandial state, a significant meal*genotype interaction (*P* = 0.046) was observed for the percentage heparin releasable binding of ^125^I-LDL in the presence of postprandial VLDL1-rich particles, with a non-significant tendency for a similar relationship for the total uptake (*P* = 0.07). Within the *APOE4* carrier group, this reflected a 38–40% and 15–17% lower percentage of heparin releasable binding and total uptake of ^125^I-LDL in the presence of VLDL1-rich particles after the SFA-rich meal compared with the UNSAT and SFA-FO meals. Differences were not evident between meals in the *APOE3/E3* group (Table 3).

In the genotype groups combined, there was a significant meal effect for the cell associated radioactivity and total uptake of ^125^I-LDL in the presence of postprandial VLDL1-rich particles (*P* = 0.05). A lower percentage total uptake was observed with TRL isolated following the SFA meal (69%) compared with the SFA-FO (79%) and UNSAT (75%) meals (*P* = 0.038).

For the VLDL2-rich fraction, in *APOE3/E4*, the percentage cell associated ^125^I-LDL was lower in the presence of particles isolated following the SFA (57%) and UNSAT (71%) meals relative to the SFA-FO (89%) (meal * genotype interaction, *P* = 0.047) which was reflected in a trend (*P* = 0.07) towards lower total ^125^I-labelled LDL uptake in the presence of SFA-enriched particles.

## Discussion

The replacement of saturated with unsaturated fats is an effective and widely recommended population dietary strategy to lower plasma total- and LDL-cholesterol. *APOE4* carrier status is associated with an ~1.5 fold higher CVD risk in some but not all studies (for meta-analysis see refs 8 and 10), moderately raised LDL-cholesterol^8^, and a greater responsiveness to dietary fatty acid composition^5^,^6^,^34^,^35^. However the mechanistic basis for these associations is largely unknown, with an unsubstantiated suggestion that an *APOE4* genotype may be associated with reduced LDLR (a core receptor which facilitates the hepatic removal of LDL and TRL from the circulation) expression^36^. Here the focus is on the interaction of lipoproteins with the LDLR. Overall, using the newly developed SPR approach, our fasting data indicates lower LDL and increased VLDL1- rich particle binding to the LDLR in *APOE4* carriers. In the postprandial state, consumption of the SFA meal resulted in enhanced binding of VLDL1-rich (overall K1 and overall K_A_) and VLDL2- rich particles (ka2) and associated strong trends towards reduced HepG2 ^125^I-labelled LDL uptake in *APOE4* carriers when these particles were co-incubated. Collectively these effects on LDLR interactions and hepatic LDL uptake provide an explanation for the higher circulating LDL-cholesterol concentrations in *APOE4* carriers, in particular in response to increased dietary SFA intakes.

Our analysis revealed *APOE genotype* to influence both stages of the LDL-LDLR binding process, with a 1.5 fold slower initial rate of association of *APOE3/E4* LDL fraction particles with the LDLR (ka1). In addition, the rate of dissociation (kd1) was also higher suggesting a lower stability of the LDL-LDLR complex in *APOE3/E4* samples, which would be predicted to result in a slower liver LDL receptor-mediated endocytosis. A slower association rate was also observed with the fasting VLDL1-rich particles from the *APOE3/E4* group, but once the VLDL1-LDLR complex was formed, it was found to be more stable and dissociated at a slower rate than the *APOE3/E3* VLDL-1 rich fraction. This finding explains the lower percentage binding of ^125^I-labelled LDL to the HepG2 cell surface (the heparin releasable fraction comprises particles bound to the LDLR, heparin-sulphate proteoglycans (HSPG) and other receptors) in the presence of the *APOE3/E4* VLDL1-rich fraction. Although a similar relationship was also observed in the HepG2 cell studies with the VLDL2-rich fraction, genotype was not shown to influence the SPR binding kinetics of this TRL fraction to the LDLR. Differences between the findings of the SPR and HepG2 cell experiments with respect to the VLDL2-rich fraction may reflect greater involvement of proteins on the cell surface (such as HSPG) for mediating the binding of this TRL fraction to the LDLR. Our SPR technique may provide some insights into this as we observed the binding characteristics of the VLDL2-rich fraction to be intermediate between the VLDL1-rich fraction and LDL, which are thought to utilize apoE and apoB-100, respectively, as a ligand for receptor-mediated uptake. Although it has been proposed that VLDL2 uses apoB-100 as a ligand^13^, our data suggest that apoE may also facilitate binding of these TRL fraction particles to the LDLR.

Our findings from the ^125^I-labelled LDL uptake studies in the presence of fasting TRL are in agreement with a previous *in vitro* study which reported a greater binding affinity of VLDL isolated from *APOE4* homozygotes in the fasted state to the LDLR^37^ but also indicate that this phenomenon is evident in *APOE4* heterozygotes. However, the total uptake of ^125^I-labelled LDL was not different between the genotype groups suggesting that raised fasting LDL-cholesterol concentrations in *APOE3/E4* individuals may be associated with, (i) delayed clearance of LDL from the circulation (as a result of the greater competition with TRL for binding to the LDLR), or (ii) preferential uptake of TRL by the liver which augments the hepatic cholesterol pools, reducing LDLR expression. Interestingly, in the present study, the fasting VLDL1-rich fraction was shown to reduce ^125^I-labelled LDL uptake to a greater extent than the VLDL2-rich fraction. This is in contrast to our previous observation, which showed the fasting VLDL2-rich fraction isolated from *E4* carriers following DHA supplementation to compete to a greater extent with ^125^I-labelled LDL for uptake, compared with VLDL1-rich fraction (6). These differences may reflect the acute nature of our current study design or the use of LDL isolated from the same *APOE* genotype group in our HepG2 competition experiments.

Although the amino acids present at positions 112 and 158 of the apoE protein are not within the receptor binding region (amino acids 136–150), it has been demonstrated that amino acid changes at these sites impact on the salt bridge formation and the 3D protein conformation which is likely to affect receptor binding activity^38^. It has also been proposed that the variable receptor binding of lipoprotein-associated apoE could be as a consequence of the two different lipid-bound states of the apoE molecule^39^. The LDLR binding domain in apoE is only recognized when the helix bundle is open, so only molecules in this conformation will act as effective ligands for this receptor^40^. This conformational change may support the two-step model seen in our sensorgrams when apoE binds to LDLR, as similar results have been reported by Nguyen *et al*., when determining the binding of apoE to VLDL particles^18^. According to this, the second step of the reaction (formation of AB*), corresponds at least in part to the rate at which the helix bundle opens. The shift from AB to AB* regulated by ka2 (when the apoE-VLDL interaction was studied by SPR) has been reported to occur faster in *APOE4* carriers than in *APOE3*/E3^18^. However, in our study, a similar ka2 constant was observed for both fasting LDL and TRL isolated from the *APOE3/E3* and *APOE3/E4* groups, suggesting that the higher binding affinity in *APOE4* carriers may reflect the conformation of APOE4 on the surface of these particles which, due to the lower stability of the helix bundle, binds to the receptor in the open conformation.

In the present study, *APOE* genotype was shown to have a significant impact on the binding properties of fasting LDL and TRL particles for the LDLR whereas meal fat composition influenced the binding kinetics of the postprandial TRL particles. Irrespective of the *APOE* genotype, a greater binding affinity for the LDLR was observed with particles isolated after the SFA-rich than UNSAT or SFA-FO meals, although this was only significant for the VLDL1-rich fraction. This finding is in agreement with an earlier *in vitro* study, in which the uptake of ^125^I-labelled LDL was reduced by the presence of apoE-rich S_f_ 60–400 particles isolated after a SFA-rich meal^14^. In the postprandial state, the metabolism of TRL particles has been shown to be altered by enrichment with ‘exchangeable’ apolipoproteins such as apoE^41^. Consistent with our previous study, the apoE: apoB ratio was also higher in the postprandial VLDL1-rich fraction after the SFA meal indicating that the test meal fat composition impacts on both the number and orientation of the apoE molecules on the particle surface^41^. Since apoE can exist in two different states when bound to lipids^40^,^42^,^43^, we speculate that apoE presents in an open helix structure on the post-meal TRL particles leading to an unmasking of the binding domain for the LDLR after the SFA relative to either SFA-FO or UNSAT meals.

Our findings suggest that the LDLR may play a more important role in the uptake of VLDL1-rich particles after a meal containing predominately SFA than UNSAT, which may reflect the effects of SFA on TRL particle composition. Individual LDL particles are reported to bind with single LDLR^44^, which are clustered together in coated pits on the surface of liver cells. The binding of large TRL enriched in apoE to one or multiple LDLR, may physically prevent the binding of LDL to the LDLR, thus reducing the number of LDL particles bound and therefore internalized and degraded by the liver^45^. The observation of a lower percentage binding of ^125^I-labelled LDL to the HepG2 cell surface in the presence of VLDL1-rich particles isolated after the SFA meal suggests that this may be a physiological phenomenon. However, the HepG2 cell studies are based on the uptake of radiolabeled LDL which can only determine the fate of the LDL particles, with no information about the uptake of the unlabeled TRL by the liver cells. This could occur via the LDLR, or they could influence LDL uptake by binding to other receptors on the cells such as the LDLR related protein, influencing cellular cholesterol concentrations and expression of the LDLR on the liver cell surface. Further work is required to determine the effects of meal fatty acid composition on the orientation of apoE on the particle surface and kinetics of binding of TRL isolated from different *APOE* genotype groups to the HSPG, LDLR and related receptors.

The development of our SPR technique has provided novel insights into the interaction of intact lipoproteins with the LDLR, with our complementary HepG2 cell studies providing a more physiological representation of the receptor-mediated uptake of LDL by liver cells in the presence of circulating TRL particles. These findings provide a mechanistic basis for the higher LDL-cholesterol associated with SFA-rich diets, and in particular in *APOE4* carriers.

## Additional Information

**How to cite this article:** Calabuig-Navarro, M. V. *et al*. A randomized trial and novel SPR technique identifies altered lipoprotein-LDL receptor binding as a mechanism underlying elevated LDL-cholesterol in *APOE4s. Sci. Rep.* **7**, 44119; doi: 10.1038/srep44119 (2017).

**Publisher's note:** Springer Nature remains neutral with regard to jurisdictional claims in published maps and institutional affiliations.

## Figures and Tables

**Table 1:** *APOE* genotype influences the binding properties of fasting LDL, VLDL1-rich, and VLDL2-rich particles to the LDLR.

SPR binding constants	Lipoprotein fraction								
	LDL			VLDL1-rich TRL			VLDL2-rich TRL		
	*APOE3/E3*	*APOE3/E4*	*P* genotype	*APOE3/E3*	*APOE3/E4*	*P* genotype	*APOE3/E3*	*APOE3/E4*	*P* genotype
ka1 × 10^5^ (M^−1^s^−1^)	21.5 ± 1.0	14.5 ± 3.9	0.77	11.3 ± 0.4	9.3 ± 0.5	0.011	10.6 ± 0.4	10.3 ± 0.4	0.71
kd1 × 10^−3^ (s^−1^)	11.7 ± 0.2	12.7 ± 0.3	0.042	6.2 ± 0.8	5.2 ± 0.5	0.28	6.0 ± 0.3	7.3 ± 1.1	0.38
K1 × 10^8^ (M^−1^)	1.8 ± 0.1	1.4 ± 0.2	0.005	2.0 ± 0.3	1.9 ± 0.2	0.37	1.8 ± 0.1	1.7 ± 0.4	0.85
ka2 × 10^−2^ (s^−1^)	0.7 ± 0.0	0.6 ± 0.0	0.22	1.5 ± 0.1	1.7 ± 0.1	0.12	1.2 ± 0.1	1.2 ± 0.1	0.92
kd2 × 10^−4^ (s^−1^)	25.4 ± 0.6	28.7 ± 1.0	0.031	11.2 ± 0.2	9.7 ± 0.4	0.013	10.6 ± 0.1	11.3 ± 0.5	0.28
K2	2.6 ± 0.1	1.7 ± 0.3	0.019	13.2 ± 0.8	17.3 ± 1.4	0.032	11.6 ± 0.8	11.1 ± 1.2	0.76
K_A_ × 10^9^ (M^−1^)	0.7 ± 0.0	0.5 ± 0.0	0.019	2.7 ± 0.4	3.3 ± 0.3	0.21	2.2 ± 0.2	1.9 ± 0.4	0.49

Values are listed as mean ± SEM for n = 5 independent SPR experiments performed using LDL, S_f_ 60–400 and S_f_ 20–60 isolated from the *APOE3/E3* and *APOE3/E4* individuals in the fasted state. Association rate constants are expressed as ka1 and ka2; dissociation rate constants kd1 and kd2. Affinity constants are expressed as K1, K2 and K_A_ for the two-state reaction model A + B ↔ AB ↔ AB*.

Differences between genotype groups were compared using the Student’s independent t-test (*P* ≤ 0.05).

**Table 2:** Effect of meal fat composition on the binding properties of postprandial VLDL1-rich and VLDL2-rich particles to the LDLR.

Fraction	SFA	SFA-FO	UNSAT	*P* meal
**VLDL1-rich**				
ka1 × 10^5^ (M^−1^s^−1^)	9.4 ± 0.5^a^	8.4 ± 0.5^b^	7.9 ± 0.6^b^	0.004
kd1 × 10^−3^ (s^−1^)	3.8 ± 0.7^a^	4.1 ± 0.6^a,b^	4.8 ± 0.1^b^	0.047
K1 × 10^8^ (M^−1^)	3.2 ± 0.6^a^	2.4 ± 0.6^b^	1.5 ± 0.1^b^	0.007
ka2 × 10^−2^ (s^−1^)	1.6 ± 0.2	1.4 ± 0.1	1.5 ± 0.1	0.67
kd2 × 10^−4^ (s^−1^)	7.9 ± 0.7	9.2 ± 0.4	8.8 ± 0.4	0.10
K2	21.1 ± 2.8	17.2 ± 0.9	18.1 ± 0.3	0.11
K_A_ × 10^9^ (M^−1^)	6.2 ± 0.8^a^	3.3 ± 0.4^b^	2.9 ± 0.2^b^	<0.001
K_D_ × 10^10^ (M)	1.8 ± 0.3^a^	3.4 ± 0.5^b^	3.7 ± 0.3^b^	<0.001
**VLDL2-rich**				
ka1 × 10^5^ (M^−1^s^−1^)	11.9 ± 1.6	10.3 ± 1.7	10.2 ± 1.0	0.28
kd1 × 10^−3^ (s^−1^)	7.8 ± 1.4	6.4 ± 1.4	6.7 ± 0.8	0.25
K1 × 10^8^ (M^−1^)	1.8 ± 0.3	1.6 ± 0.4	1.8 ± 0.3	0.59
ka2 × 10^−2^ (s^−1^)	1.7 ± 0.2^a^	1.3 ± 0.3^a,b^	1.3 ± 0.2^b^	0.011
kd2 × 10^−4^ (s^−1^)	8.9 ± 0.6	9.3 ± 0.4	10.5 ± 1.0	0.46
K2	20.7 ± 3.7	42.1 ± 24.8	13.3 ± 2.1	0.41
K_A_ × 10^9^ (M^−1^)	3.8 ± 0.6	3.0 ± 0.5	2.4 ± 0.4	0.09
K_D_ × 10^10^ (M)	3.7 ± 0.8	4.6 ± 1.0	5.9 ± 1.5	0.265

Values represent mean ± SEM for n = 10 independent SPR samples for the VLDL1 and VLDL2-rich fractions, respectively.

Abbreviations: SFA, saturated fatty acid meal; SFA-FO, SFA meal with fish oil; UNSAT, unsaturated fatty acid meal.

Data for the two genotype groups combined were analyzed using a one-way repeated measures ANOVA followed by Student’s t test with Bonferroni correction (P < 0.017). Values within each row with different superscript letters are significantly different.

**Table 3:** Summary measures for the uptake of ^125^I-labelled LDL by HepG2 cells in the presence of *ex vivo* VLDL1 and VLDL2-rich TRL fractions isolated from fasting and postprandial plasma samples.

	Fasting			Postprandial						*P* for ANOVA		
	*APOE3/E3*	*APOE3/E4*	*P*	*APOE3/E3*			*APOE3/E4*			Meal	Genotype	Meal × genotype
				SFA	SFA-FO	UNSAT	SFA	SFA-FO	UNSAT			
**VLDL1-rich**												
Heparin^1^	108.2 ± 3.5	49.8 ± 12.1	0.001	63.7 ± 10.0	65.5 ± 9.2	66.1 ± 11.4	51.2 ± 7.3^a^	82.6 ± 13.4^b^	84.8 ± 18.5^b^	0.023	0.63	0.046
Cell associated^1^	54.9 ± 7.4	59.3 ± 5.9	0.67	77.6 ± 7.8	84.2 ± 8.1	71.8 ± 6.3	66.0 ± 17.8	81.7 ± 17.6	70.4 ± 8.7	0.036	0.74	0.48
Deg. Products^1^	88.2 ± 8.2	81.2 ± 3.9	0.51	59.9 ± 13.4	66.8 ± 13.3	73.8 ± 15.9	77.8 ± 21.8	89.6 ± 19.3	94.0 ± 20.1	0.17	0.49	0.43
Total uptake^1^	65.3 ± 5.6	68.5 ± 2.4	0.89	70.1 ± 8.7	76 ± 8.8	69.1 ± 9.8	67.6 ± 8.8^a^	81.9 ± 8.4^b^	79.9 ± 9.8^b^	0.053	0.89	0.07
**VLDL2-rich**												
Heparin	109.2 ± 9.9	75.1 ± 8.3	0.038	61.8 ± 12.8	58.1 ± 10.6	55.0 ± 11.0	62.1 ± 10.3	91.1 ± 34.7	86.9 ± 21.0	0.39	0.37	0.16
Cell assoc	77.0 ± 6.8	88.6 ± 5.9	0.25	54.8 ± 11.7	57.0 ± 13.7	49.9 ± 9.0	56.9 ± 13.8^a^	89.0 ± 21.9^b^	70.5 ± 16.9^a^	0.019	0.38	0.047
Deg products	92.3 ± 7.7	70.9 ± 7.5	0.32	54.4 ± 8.9	44.8 ± 6.3	50.4 ± 6.8	60.5 ± 16.9	94.6 ± 44.7	82.1 ± 29.3	0.12	0.33	0.07
Total uptake	81.9 ± 6.7	81.8 ± 6.3	0.32	54.9 ± 9.5	51.9 ± 10.5	50.6 ± 8.0	55.7 ± 8.2	88.1 ± 26	73 ± 18.2	0.13	0.32	0.07

Data are mean ± SEM for the percentage change in heparin releasable binding, cell associated radioactivity, degradation products and total uptake of ^125^I-labelled LDL after 5 h in the presence of VLDL1 and VLDL2-rich fractions, compared with ^125^I-labelled LDL in the absence of TRL. Each experiment was performed in duplicate and represents n = 5 independent experiments for *APOE3/E3* and n = 4 independent experiments for *APOE3/E4* group.

Heparin- refers to the heparin releasable, cell surface bound ^125^I-LDL, Cell associated- refers to the ^125^I-LDL which has been internalized into the cell, Deg. products- refers to the degradation products which have been released by the cell back into the medium, Total uptake is the sum of the cell surface bound (heparin), cell associated and degradation products.

The absolute values for the total uptake of ^125^I-labelled LDL in the absence of TRL was 948 ± 137 ng/mg cell protein (heparin releasable binding 40 ± 5 ng/mg cell protein, cell associated radioactivity 587 ± 81 ng/cell protein and degradation products 323 ± 37 ng/cell protein) and 881 ± 127 ng/cell protein (heparin releasable binding 38 ± 5 ng/cell protein, cell associated radioactivity 480 ± 76 ng/cell protein and degradation products 363 ± 53 ng/cell protein) for *APOE3/E3* and *APOE3/E4* LDL, respectively.

In the fasting state, differences between genotype groups were compared using the Student’s independent t-test (P ≤ 0.05). For the TRLs isolated after the saturated fatty acid meal (SFA), SFA meal with fish oil (SFA-FO) and unsaturated fatty acid meal (UNSAT), data were analyzed using a mixed factor ANOVA. When the meal*genotype interaction were significant, one way repeated measures ANOVA followed by Student’s t test with Bonferroni correction (P < 0.017) were used to determine differences between meals within each genotype group. Values with different superscript letters within each row are significantly different.
